# Association between milk consumption and child growth for children aged 6–59 months

**DOI:** 10.1038/s41598-020-63647-8

**Published:** 2020-04-21

**Authors:** Christine Herber, Lisa Bogler, S. V. Subramanian, Sebastian Vollmer

**Affiliations:** 1 Department of Economics and Centre for Modern Indian Studies, University of Goettingen, Goettingen, Germany; 2 Department of Social and Behavioral Sciences, Harvard T.H. Chan School of Public Health, Boston, USA; 3 Harvard Center for Population and Development Studies, Cambridge, MA USA

**Keywords:** Nutrition, Health policy

## Abstract

Apart from high levels of energy, proteins, micro- and macronutrients, milk contains calcium and the insulin-like growth factor-1 that are of major relevance for children’s development and growth. Using Demographic and Health Survey data between 1990 and 2017 with information on milk consumption and anthropometric measurements from all low- and middle-income countries available, we investigate whether milk consumption in childhood is associated with stunting, wasting, and underweight. We specify logistic regression models and adjust for a range of covariates and fixed effects on the primary sampling unit level. We analyze heterogeneity in the association by wealth quintiles and age groups and present country-specific estimates. The final samples for wasting, underweight and stunting include 668.463, 693.376, and 673.177 observations of children aged 6 to 59 months, respectively. Our results suggest that milk consumption is associated with a reduced probability of being underweight of 1.4 percentage points (95% confidence interval −0.02, −0.01) and a reduced probability of being stunted of 1.9 percentage points (95% confidence interval −0.02, −0.01). The association for wasting is not robust. The association is stronger for children from wealthier households, which might indicate that milk consumption is a proxy for better overall nutrition or socio-economic status.

## Introduction

Milk consumption in childhood has long been assumed to be beneficial for growth, resulting in the “milk hypothesis” suggested by Bogin^1^. While children are ideally exclusively breastfed during the first six months of life, consumption of nonhuman milk after the age of six months may stimulate growth in the following years^2–4^. This association may be due to the nutritious content of milk. Apart from high levels of energy, proteins and micro- and macronutrients, milk contains calcium and the insulin-like growth factor-1 (IGF-1) that are of major relevance for children’s development and growth^3,5^.

In one of the first studies on milk consumption and growth in 1928, Boyd Orr estimated an increase of 20% in height and weight for Scottish children aged 5 to 14 years who consumed milk in addition to their normal diet for seven months in comparison to children who did not^6^. For low- and middle-income countries, few studies focusing on milk consumption and growth are available. Early studies from 1970 and 1977 found significant increases in weight and height for children consuming milk compared to a control group in a school milk intervention in New Guinea^7,8^. More recent evidence comes from a number of RCTs in Kenya and Vietnam^9,10^ and cross-sectional studies in Cambodia and Uganda^11,12^. Recent studies that look at livestock ownership in Bangladesh and Kenya also assess the association between milk consumption and child growth^13,14^.

Although results in general point towards a positive effect of milk consumption on child growth, evidence remains mixed when looking at different indicators. For example, while Grillenberger *et al*. found significant changes for the weight-for-height z-score but not for the height-for-age z-score in an RCT with Kenyan schoolchildren^9^, Nhung *et al*. found a significant change in the height-for-age z-score but not in the weight-for-height z-score for schoolchildren in Vietnam^10^. Moreover, these studies are country-specific, differ in their measurement of milk consumption, and focus on different age groups, ranging from children in their first five years of life to children aged 6 to 14 years. These factors reduce comparability.

Given the small number and the country focus of studies that analyze the association between milk consumption and child growth in low- and middle-income countries, we aimed to investigate the cross-country association for all low- and middle-income countries for which comparable data is available. We present estimations for the pooled sample of 68 countries as well as country-specific estimates. Our analysis focused on children aged 6 to 59 months and used three anthropometric outcomes. These outcomes measure both child growth and undernutrition continuously and categorically (z-scores and resulting moderate and severe forms of wasting, underweight, and stunting). Adding a further layer to the analysis, we explored heterogeneous effects by age and wealth, a novel contribution to the literature.

## Methods

### Data

In this paper we used data of the Demographic and Health Surveys (DHS), administered by ICF International (available at https://dhsprogram.com/data/available-datasets.cfm). In seven survey rounds since 1984, the DHS Program has collected nationally representative data in low- and middle-income countries. This paper used data from the second phase onwards from the woman’s individual recode data, in which women in reproductive age, typically between 15 and 49 years, were asked for information on all children ever born to them. A multistage stratified sampling design was applied for the within-country selection of households. For each country, regions were defined, within which the population was stratified into urban and rural. For each stratified area, enumeration areas were randomly drawn and denoted as primary sampling units (PSUs). Selection of PSUs was based on a probability proportional to size, in this case the number of households. Within each PSU, all households were listed from the most recent population census. Applying an equal probability systematic sampling, around 25 households in each PSU were selected for an interview. Survey final reports provide detailed sampling plans for each country and survey^15^. Weights for the calculation of nationally representative statistics are provided with the survey data. The Institutional Review Board of ICF International and the relevant human subject committees in each country approved the data collection procedures. The methods used in this analysis were performed in accordance with the relevant guidelines and regulations. Respondents or their parents/legal guardian provided informed consent for study participation. As the study was based on an anonymous public use data set with no identifiable information on the study participants, it is exempt from ethics committee approval at the University of Göttingen.

### Outcomes

We analyzed milk consumption with respect to three anthropometric outcomes, measuring both child growth and undernutrition continuously and categorically (z-scores and resulting moderate and severe forms of wasting, underweight, and stunting). In most DHS, anthropometric measures were taken for children under the age of five years at the point of the interview. Z-scores were calculated as the difference of each child’s height or length (hereafter referred to as height) and weight to the age- and sex-specific median established within the WHO Multicentre Growth Reference Study in 2006, divided by the standard deviation of the respective reference^16^. Z-scores are defined as continuous outcomes. Categorical forms of undernutrition are binary variables either indicating the presence or absence of the outcome for each child. A child is classified as wasted, underweight, or stunted, if the respective z-score is more than two standard deviations below the reference mean. The underlying measure for wasting is weight-for-height, underweight refers to weight-for-age, and stunting refers to height-for-age z-scores. Children are classified as severely undernourished, if the respective z-score falls below three standard deviations of the reference mean. Observations with biologically implausible high or low z-scores were excluded from the sample (weight-for-height z-scores < −5 | > 5, height-for-age z-scores < −6 | > 6, weight-for-age z-scores < −6 | > 5)^16^. All z-scores were calculated using the *igrowup*_*stata* program, provided by the software *WHO Anthro* (Version 3.2.2)^17^.

### Exposure and covariates

To analyze the association between milk consumption and child growth status, we defined the exposure as the consumption of tinned, powdered or any fresh milk during the 24 hours preceding the interview. We excluded the first DHS-phase, surveys before 1990, from the calculation. In all subsequent survey rounds the exposure was reported. Our exposure variable was a composite binary variable equal to 1, if any of the phase-specific variables indicated that the child was given milk at least once during the day or night before the interview. We assumed that the milk consumption during the last 24 hours allows for inference on the general milk consumption of the child, although there are several weaknesses to that assumption^18^. Variables indicating reported milk consumption during the seven days preceding the interviews were not included in the estimation, as they only provided data for few observations of the third to fifth survey round.

At child-level, both adjusted model specifications controlled for sex, age in six-month intervals, birth order, whether the child was part of a multiple birth, whether it is currently breastfed, and the duration of breastfeeding in months. In an alternative specification, we also controlled for birth weight. However, as this considerably reduced the sample size, it was not included in the main analysis. To account for overall feeding practices, a binary control was defined, indicating whether the child’s diet during the 24 hours before the interview consisted of a minimum acceptable diet, which was fulfilled when both a minimum dietary diversity and a minimum meal frequency were given^19^. The mother’s educational level was included as a categorical variable, indicating the highest educational level attained (no education, primary, secondary, or higher education). The partner’s education was not controlled for to avoid the exclusion of mothers who were not in a partnership at the time of the interview. The maternal partnership status at the time of the interviews was accounted for. Additionally, the age of the mother at birth was included in five-year intervals. Rural and urban location was taken into account by the inclusion of fixed effects on the PSU-level. An asset index of observed household assets was calculated using principal-component analysis to define asset-based country- and survey-specific wealth quintiles. Calculations were based on the assets radio, television, bicycle, car, motorcycle, refrigerator, phone, electricity, piped drinking water, flush toilet, floor material, wall material, and roof material. Households with information on less than 90% of underlying assets were excluded.

### Statistical analysis

Three model specifications were estimated. The first model did not include covariates. The second specification included all previously described covariates (except minimum acceptable diet) and fixed effects on the PSU-level. The third model specification additionally controlled for a minimum acceptable diet. The inclusion of the minimum acceptable diet led to all surveys of DHS phase 2 being dropped. For the categorical outcomes (moderate and severe forms of wasting, underweight and stunting), the unadjusted model was based on logistic regression, the second and third specifications were conditional logistic models to account for the inclusion of fixed effects. Associations were reported as average marginal effects (AME). All estimates were unweighted, descriptive statistics were population weighted. Continuous outcomes, the z-scores, were estimated using multilevel linear regression. Standard errors were clustered on the PSU-level to account for the hierarchical data structure. Exceptions were standard errors of continuous outcomes in adjusted models, which were clustered on the country level for computational reasons. We present main effects among all wealth quintiles and ages. In addition, we present the estimated association between milk consumption and child growth differentiated by wealth quintiles and age categories (6–23 months, 24–59 months). All estimates were calculated using Stata 14 (StataCorp LP).

## Results

### Sample description

**Figure 1:**
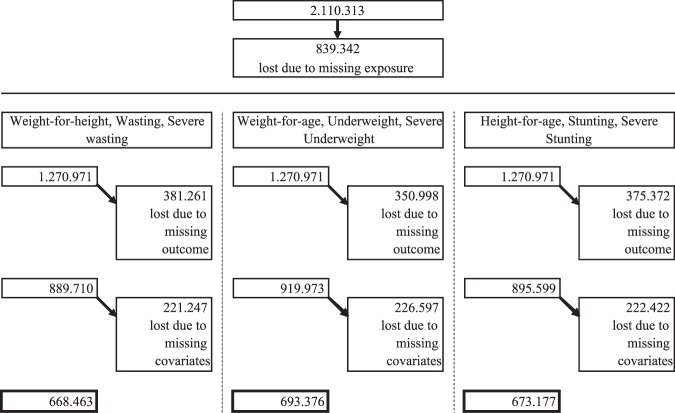
Sample selection.

Our initial sample consisted of more than 2 million children between 6 and 59 months of age that were alive and living permanently in the respective household at the time of the interview. Figure 1 illustrates the different steps of the sample selection. 839.342 observations had to be excluded, because the main explanatory variable, information on milk consumption during the past 24 hours, was missing or incalculable. This led to a sample of 1.270.971 children from 234 DHS in 73 low- and middle-income countries. Included surveys were conducted between 1990 and 2017. Further observations were lost due to missing outcome variables and main covariates, excluding minimum acceptable diet. The final number of observations included in estimating the second specification is 668.463 for weight-for-length z-scores, moderate and severe wasting, 693.376 for weight-for-age z-score, moderate and severe underweight, and 673.177 for height-for-age z-score, moderate and severe stunting.

### Findings

Table 1 presents population weighted descriptive statistics of the prevalence of undernutrition. Further sample characteristics are presented in the supplementary material. The overall prevalence of wasting in our sample (reduced for observations with missing exposure and outcome) was 12.4%, while the prevalence of underweight was 30.0% and that of stunting was 42.3%. Not controlling for any covariates, the prevalence of all indicators of undernutrition was higher among the sample of children that did not consume milk during the 24 hours preceding the interview than among the sample of children that did consume milk (*P* < 0.001 for all indicators). 39% of our sample consumed milk during the 24 hours preceding the interview, ranging from 29% in the poorest quintile to 53% in the richest quintile, a statistically significant difference in milk consumption. Milk consumption and the prevalence of undernutrition vary widely across countries (see Supplementary Table S2). While only 3% of children consumed milk in Mozambique (2011), 97% consumed milk in Kazakhstan (1999). The prevalence of stunting and underweight both decreased over time, but there was no similar pattern for the prevalence of wasting and milk consumption (see Supplementary Figures S1–S4).

**Table 1:** Population Weighted Descriptive Statistics and Prevalence of Undernutrition.

Outcome variable	N	Overall outcome prevalence (% of N)	Prevalence among children consuming milk (%)	Prevalence among children not consuming milk (%)
Wasting	889,710	12.37	11.25	13.11
Severe wasting	889,710	4.25	3.27	4.95
Underweight	919,973	29.97	26.51	32.23
Severe underweight	919,973	11.82	10.00	13.02
Stunting	895,599	42.34	38.01	45.17
Severe stunting	895,599	20.87	17.65	22.99

Underlying samples are reduced for observations with missing exposure and outcome.

Table 2 presents estimation results of the association between milk consumption and undernutrition across all wealth quintiles and age groups. We expected to see positive coefficients for the continuous z-scores as outcomes. In fact, the association of milk consumption with the three z-scores was positive and highly significant in the unadjusted model. However, the size of the coefficients decreased and significance weakened when controlling for covariates and adding fixed effects. In the third model, milk consumption was associated with an increased height-for-age z-score of 0.06 points (*P* < 0.001) and an increased weight-for-age z-score of 0.03 points (*P* = 0.005), but not with a change in the weight-for-height z-score. For the binary outcomes capturing moderate and severe undernutrition we expected to see negative coefficients as milk consumption was hypothesized to reduce the prevalence of undernutrition. These associations seemed to be more robust across the specifications, with the exception of wasting. Controlling for all covariates and including fixed effects, milk consumption was associated with a reduced probability of being underweight by 1.4 percentage points (*P* < 0.001) and of being stunted by 1.9 percentage points (*P* < 0.001). This finding was similar for severe undernutrition, suggesting a reduced probability of being severely underweight and severely stunted by 1.7 percentage points (both *P* < 0.001). The associations with wasting and severe wasting were not statistically significant and very close to zero.

**Table 2:** Association Between Milk Consumption and Undernutrition across all Wealth Quintiles and Age Groups.

z-scores	Unadjusted			Adjusted (2)			Adjusted (3)		
	Weight-for-height	Weight-for-age	Height-for-age	Weight-for-height	Weight-for-age	Height-for-age	Weight-for-height	Weight-for-age	Height-for-age
Milk consumption									
	0.1566^***^	0.3039^***^	0.3063^***^	0.0062	0.0540^***^	0.0809^***^	−0.0051	0.0297^**^	0.0581^***^
*CI 95%*	[0.15,0.16]	[0.30,0.31]	[0.30,0.32]	[−0.02,0.03]	[0.03,0.08]	[0.06,0.10]	[−0.03,0.02]	[0.01,0.05]	[0.03,0.08]
*P*	0.0000	0.0000	0.0000	0.5678	0.0000	0.0000	0.6544	0.0051	0.0000
*N*	889,710	919,973	895,599	668,463	693,376	673,177	487,289	500,389	490,227
**Moderate undernutrition**	**Wasting**	**Underweight**	**Stunting**	**Wasting**	**Underweight**	**Stunting**	**Wasting**	**Underweight**	**Stunting**
Milk consumption (AMEs)									
	−0.0179^***^	−0.0595^***^	−0.0921^***^	−0.0067^*^	−0.0220^***^	−0.0264^***^	−0.0009	−0.0140^***^	−0.0194^***^
*CI 95%*	[−0.02,−0.02]	[−0.06,−0.06]	[−0.09,−0.09]	[−0.01,−0.00]	[−0.03,−0.02]	[−0.03,−0.02]	[−0.01,0.01]	[−0.02,−0.01]	[−0.02,−0.01]
*P*	0.0000	0.0000	0.0000	0.0137	0.0000	0.0000	0.7910	0.0000	0.0000
*N*	889,710	919,973	895,599	321,811	462,182	541,362	203,459	301,037	371,499
**Severe undernutrition**	**Sev. Wasting**	**Sev. Underweight**	**Sev. Stunting**	**Sev. Wasting**	**Sev. Underweight**	**Sev. Stunting**	**Sev. Wasting**	**Sev. Underweight**	**Sev. Stunting**
Milk consumption (AMEs)									
	−0.0074^***^	−0.0237^***^	−0.0565^***^	−0.0055	−0.0254^***^	−0.0238^***^	−0.0032	−0.0173^***^	−0.0173^***^
*CI 95%*	[−0.01,−0.01]	[−0.03,−0.02]	[−0.06,−0.05]	[−0.01,0.00]	[−0.03,−0.02]	[−0.03,−0.02]	[−0.01,0.01]	[−0.03,−0.01]	[−0.02,−0.01]
*P*	0.0000	0.0000	0.0000	0.1735	0.0000	0.0000	0.5044	0.0001	0.0000
*N*	889,710	919,973	895,599	184,153	295,216	421,668	103,625	172,319	274,003

Covariates in adjusted models: Sex of child, age of child, birth order, part of a multiple birth, child currently breastfed, duration of breastfeeding in months, education of mother, age of mother at birth, current partnership status of mother, wealth quintiles. Third specification additionally controls for a minimum acceptable diet. All adjusted models include fixed effects on the PSU level. AMEs are based on logistic regression in the first specification and on conditional logistic regression in the second and third specification. Standard errors are clustered at the PSU-level. Standard errors of adjusted models for continuous outcomes are clustered on the country level. Estimates are unweighted.

^*^p < 0.05, ^**^p < 0.01, ^***^p < 0.001.

Next, we split the sample to analyze heterogeneities by wealth and age. Findings by wealth quintiles are presented in Table 3. In the unadjusted model we found larger coefficients for children in the wealthiest quintile compared to the poorest quintile on all outcome variables. The estimates were again attenuated when controlling for covariates and adding fixed effects. In the third specification we found no significant associations with any outcomes for children in the poorest quintile. For children in the wealthiest quintile, milk was associated with an increased weight-for-age z-score by 0.05 points (*P* = 0.006) and the height-for-age z-score by 0.11 points (*P* < 0.001). Using the binary indicators for undernutrition, we found negative effects on moderate and severe underweight and stunting for children in the wealthiest quintile. Milk consumption was associated with a reduced probability of being underweight or stunted by 2.1 and 3.4 percentage points, respectively (*P* < 0.001). The estimates for severe underweight and stunting were −2.2 and −2.6 percentage points (*P* = 0.013 and *P* < 0.001). Estimates for the weight-for-height z-score and wasting indicators were not statistically significantly for any wealth quintile.

**Table 3:** Association Between Milk Consumption and Undernutrition in the Poorest and Wealthiest Quintile.

z-scores	Unadjusted (1)			Adjusted (2)			Adjusted (3)		
	Weight-for-height	Weight-for-age	Height-for-age	Weight-for-height	Weight-for-age	Height-for-age	Weight-for-height	Weight-for-age	Height-for-age
Milk consumption									
Poorest quintile									
	−0.0125	−0.0640^***^	−0.0937^***^	−0.0026	0.0320^**^	0.0431^**^	0.0098	0.0080	0.0241
*CI 95%*	[−0.03,0.01]	[−0.08,−0.05]	[−0.11,−0.07]	[−0.02,0.02]	[0.01,0.05]	[0.02,0.07]	[−0.02,0.04]	[−0.02,0.04]	[−0.01,0.06]
*P*	0.1744	0.0000	0.0000	0.7812	0.0053	0.0027	0.4624	0.5756	0.1946
Wealthiest quintile									
	0.3115^***^	0.6667^***^	0.7234^***^	0.0198	0.0904^***^	0.1288^***^	−0.0081	0.0477^**^	0.1074^***^
*CI 95%*	[0.30,0.32]	[0.65,0.68]	[0.71,0.74]	[−0.02,0.06]	[0.04,0.14]	[0.10,0.16]	[−0.04,0.03]	[0.01,0.08]	[0.07,0.14]
*P*	0.0000	0.0000	0.0000	0.3297	0.0003	0.0000	0.6619	0.0057	0.0000
*N*	843,326	872,435	849,017	668,463	693,376	673,177	487,289	500,389	490,227
**Moderate Undernutrition**	**Wasting**	**Underweight**	**Stunting**	**Wasting**	**Underweight**	**Stunting**	**Wasting**	**Underweight**	**Stunting**
Milk consumption (AMEs)									
Poorest quintile									
	0.0046^**^	0.0232^***^	0.0217^***^	−0.0105	−0.0179^***^	−0.0146^***^	−0.0130	−0.0058	−0.0060
*CI 95%*	[0.00,0.01]	[0.02,0.03]	[0.02,0.03]	[−0.02,0.00]	[−0.03,−0.01]	[−0.02,−0.01]	[−0.03,0.00]	[−0.02,0.01]	[−0.01,0.00]
*P*	0.0077	0.0000	0.0000	0.0756	0.0006	0.0004	0.0534	0.3222	0.1869
Wealthiest quintile									
	−0.0383^***^	−0.1362^***^	−0.1990^***^	−0.0117^*^	−0.0332^***^	−0.0431^***^	−0.0016	−0.0211^***^	−0.0336^***^
*CI 95%*	[−0.04,−0.04]	[−0.14,−0.13]	[−0.20,−0.20]	[−0.02,−0.00]	[−0.04,−0.02]	[−0.05,−0.04]	[−0.01,0.01]	[−0.03,−0.01]	[−0.04,−0.02]
*P*	0.0000	0.0000	0.0000	0.0156	0.0000	0.0000	0.7953	0.0002	0.0000
*N*	843,326	872,435	849,017	321,811	462,182	541,362	203,459	301,037	371,499
**Severe Undernutrition**	**Sev. Wasting**	**Sev. Underweight**	**Sev. Stunting**	**Sev. Wasting**	**Sev. Underweight**	**Sev. Stunting**	**Sev. Wasting**	**Sev. Underweight**	**Sev. Stunting**
Milk consumption (AMEs)									
Poorest quintile									
	0.0024^*^	0.0170^***^	0.0155^***^	0.0098	−0.0078	−0.0144^**^	0.0001	−0.0040	−0.0068
*CI 95%*	[0.00,0.00]	[0.01,0.02]	[0.01,0.02]	[−0.01,0.03]	[−0.02,0.01]	[−0.02,−0.00]	[−0.02,0.02]	[−0.02,0.01]	[−0.02,0.00]
*P*	0.0206	0.0000	0.0000	0.2862	0.2709	0.0039	0.9930	0.6171	0.2239
Wealthiest quintile									
	−0.0156^***^	−0.0612^***^	−0.1220^***^	−0.0165^*^	−0.0401^***^	−0.0381^***^	−0.0180	−0.0219^*^	−0.0255^***^
*CI 95%*	[−0.02,−0.01]	[−0.06,−0.06]	[−0.12,−0.12]	[−0.03,−0.00]	[−0.05,−0.03]	[−0.05,−0.03]	[−0.04,0.00]	[−0.04,−0.00]	[−0.04,−0.01]
*P*	0.0000	0.0000	0.0000	0.0273	0.0000	0.0000	0.0518	0.0126	0.0003
*N*	843,326	872,435	849,017	184,153	295,216	421,668	103,625	172,319	274,003

Covariates in adjusted models: Sex of child, age of child, birth order, part of a multiple birth, child currently breastfed, duration of breastfeeding in months, education of mother, age of mother at birth, current partnership status of mother, wealth quintiles. Third specification additionally controls for a minimum acceptable diet. All adjusted models include fixed effects on the PSU level. AMEs are based on logistic regression in the first specification and on conditional logistic regression in the second and third specification. Standard errors are clustered at the PSU-level. Standard errors of adjusted models for continuous outcomes are clustered on the country level. Estimates are unweighted.

^*^p < 0.05, ^**^p < 0.01, ^***^p < 0.001.

The results of our analysis by age group are shown in Table 4. As for the estimations presented above, controlling for covariates and adding fixed effects reduced the size and significance of coefficients in most cases. The weight-for-height z-score and weight-for-age z-score were positively associated with milk consumption for children aged 6 to 23 months, while there was no association for the height-for-age z-score. For children aged 24 to 59 months, we found a negative association with the weight-for-height z-score, no association with the weight-for-age z-score and a positive association with the height-for-age z-score. A child aged 24 to 59 months consuming milk had a height-for-age z-score higher by 0.14 points (*P* < 0.001) compared to a child in the same age range that does not consume milk. Moderate and severe underweight were affected similarly in both age groups. Milk consumption was associated with a reduced probability of being underweight by 1.5 and 1.2 percentage points for the younger and older children (*P* < 0.001 and *P* = 0.006), and a reduced probability of being severely underweight of 1.6 and 2.1 percentage points for the younger and older children (*P* = 0.002 for both). The estimates for stunting were small and insignificant for the younger age group, while a significant association was found for the older age group. The results for wasting suggest a higher probability for children aged 24 to 59 months who consume milk.

**Table 4:** Association Between Milk Consumption and Undernutrition in different Age Categories.

z-scores	Unadjusted (1)			Adjusted (2)			Adjusted (3)		
	Weight-for-height	Weight-for-age	Height-for-age	Weight-for-height	Weight-for-age	Height-for-age	Weight-for-height	Weight-for-age	Height-for-age
Milk consumption									
6–23 months of age									
	0.0312^***^	0.3275^***^	0.4663^***^	0.0809^***^	0.0727^***^	0.0260	0.0692^***^	0.0481^***^	0.0028
*CI 95%*	[0.02,0.04]	[0.32,0.34]	[0.46,0.48]	[0.05,0.11]	[0.05,0.10]	[−0.01,0.06]	[0.04,0.10]	[0.02,0.07]	[−0.03,0.03]
*P*	0.0000	0.0000	0.0000	0.0000	0.0000	0.1184	0.0000	0.0002	0.8622
24–59 months of age									
	0.2794^***^	0.2807^***^	0.1501^***^	−0.0841^***^	0.0314^*^	0.1469^***^	−0.1129^***^	0.0028	0.1384^***^
*CI 95%*	[0.27,0.29]	[0.27,0.29]	[0.14,0.16]	[−0.12,−0.05]	[0.00,0.06]	[0.12,0.17]	[−0.15,−0.07]	[−0.03,0.03]	[0.11,0.17]
*P*	0.0000	0.0000	0.0000	0.0000	0.0257	0.0000	0.0000	0.8646	0.0000
*N*	889,710	919,973	895,599	668,463	693,376	673,177	487,289	500,389	490,227
**Moderate Undernutrition**	**Wasting**	**Underweight**	**Stunting**	**Wasting**	**Underweight**	**Stunting**	**Wasting**	**Underweight**	**Stunting**
Milk consumption (AMEs)									
6–23 months of age									
	0.0091^***^	−0.0542^***^	−0.1178^***^	−0.0189^***^	−0.0245^***^	−0.0067^*^	−0.0104^**^	−0.0150^***^	0.0005
*CI 95%*	[0.01,0.01]	[−0.06,−0.05]	[−0.12,−0.11]	[−0.03,−0.01]	[−0.03,−0.02]	[−0.01,−0.00]	[−0.02,−0.00]	[−0.02,−0.01]	[−0.01,0.01]
*P*	0.0000	0.0000	0.0000	0.0000	0.0000	0.0101	0.0097	0.0000	0.8670
24–59 months of age									
	−0.0486^***^	−0.0683^***^	−0.0693^***^	0.0133^***^	−0.0186^***^	−0.0466^***^	0.0221^***^	−0.0122^**^	−0.0444^***^
*CI 95%*	[−0.05,−0.05]	[−0.07,−0.07]	[−0.07,−0.07]	[0.01,0.02]	[−0.02,−0.01]	[−0.05,−0.04]	[0.01,0.03]	[−0.02,−0.00]	[−0.05,−0.04]
*P*	0.0000	0.0000	0.0000	0.0003	0.0000	0.0000	0.0000	0.0062	0.0000
*N*	889,710	919,973	895,599	321,811	462,182	541,362	203,459	301,037	371,499
**Severe undernutrition**	**Sev. Wasting**	**Sev. Underweight**	**Sev. Stunting**	**Sev. Wasting**	**Sev. Underweight**	**Sev. Stunting**	**Sev. Wasting**	**Sev. Underweight**	**Sev. Stunting**
Milk consumption (AMEs)									
6–23 months of age									
	0.0033^***^	−0.0195^***^	−0.0695^***^	−0.0139^**^	−0.0270^***^	−0.0043	−0.0102	−0.0159^**^	0.0037
*CI 95%*	[0.00,0.00]	[−0.02,−0.02]	[−0.07,−0.07]	[−0.02,−0.00]	[−0.04,−0.02]	[−0.01,0.00]	[−0.02,0.00]	[−0.03,−0.01]	[−0.00,0.01]
*P*	0.0000	0.0000	0.0000	0.0062	0.0000	0.2027	0.0827	0.0015	0.3293
24–59 months of age									
	−0.0200^***^	−0.0305^***^	−0.0494^***^	0.0093	−0.0234^***^	−0.0425^***^	0.0139^*^	−0.0206^**^	−0.0430^***^
*CI 95%*	[−0.02,−0.02]	[−0.03,−0.03]	[−0.05,−0.05]	[−0.00,0.02]	[−0.03,−0.01]	[−0.05,−0.04]	[0.00,0.03]	[−0.03,−0.01]	[−0.05,−0.03]
*P*	0.0000	0.0000	0.0000	0.0944	0.0000	0.0000	0.0480	0.0018	0.0000
*N*	889,710	919,973	895,599	184,153	295,216	421,668	103,625	172,319	274,003

Covariates in adjusted models: Sex of child, age of child, birth order, part of a multiple birth, child currently breastfed, duration of breastfeeding in months, education of mother, age of mother at birth, current partnership status of mother, wealth quintiles. Third specification additionally controls for a minimum acceptable diet. All adjusted models include fixed effects on the PSU level. AMEs are based on logistic regression in the first specification and on conditional logistic regression in the second and third specification. Standard errors are clustered at the PSU-level. Standard errors of adjusted models for continuous outcomes are clustered on the country level. Estimates are unweighted.

^*^p < 0.05, ^**^p < 0.01, ^***^p < 0.001.

**Figure 2:**
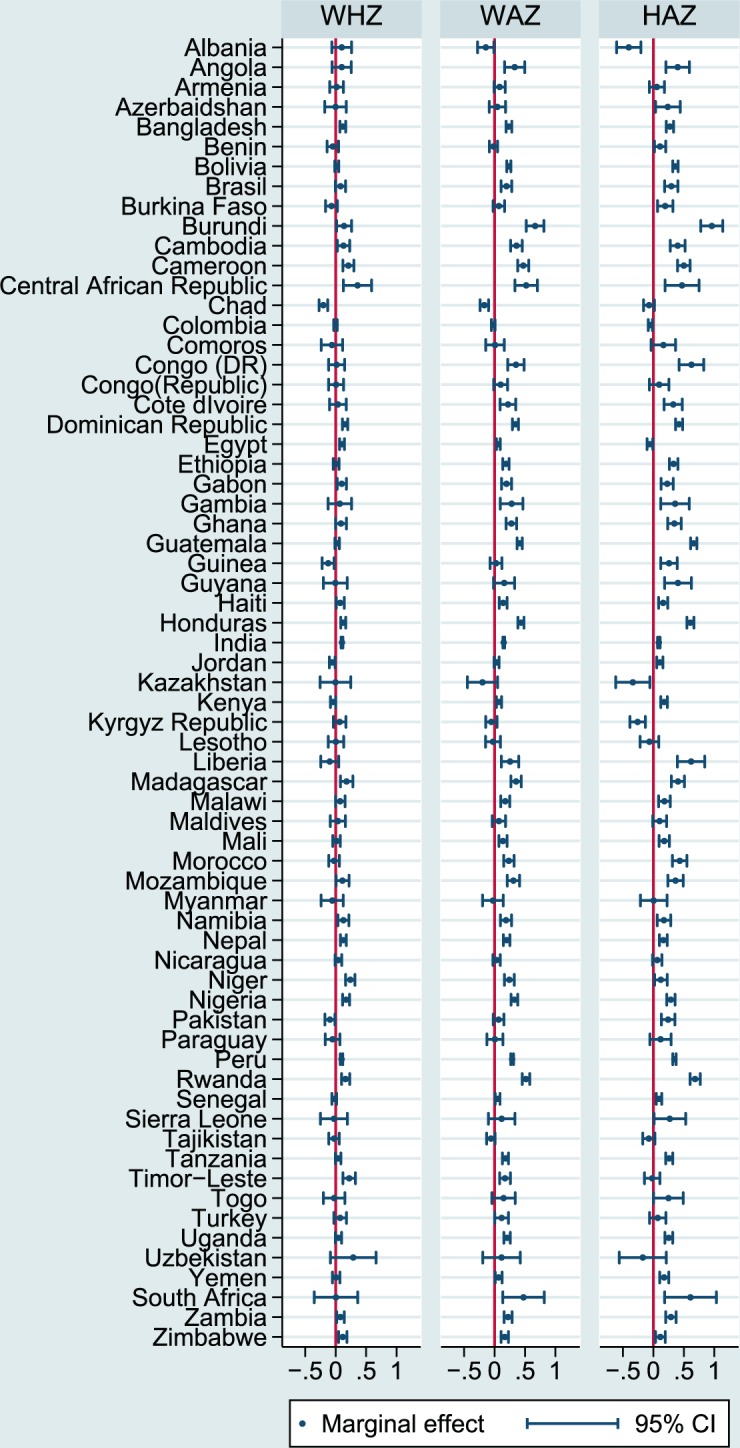
Country-Specific Association between Milk Consumption and Undernutrition. All estimates are marginal effects based on linear regressions for the first model specification (unadjusted). Standard errors are clustered on the PSU level.

Figure 2 presents country-specific estimates of the association between milk consumption and the continuous z-scores. We found large variation in the size of coefficients between countries for all three outcomes. While the association was positive for most, significantly negative coefficients were found for individual countries. In most cases, the association was strongest for the height-for-age z-score and weakest for the weight-for-height z-score. The results were similar but attenuated when controlling for covariates (see Supplementary Figures S5 and S6). Due to the lower number of observations per country, estimations were less precise in these adjusted models.

## Discussion

Using a large sample of children in low- and middle-income countries, we assessed the association between milk consumption and child undernutrition and growth. We found that milk consumption was associated with increased weight-for-age and height-for-age z-scores and reduced the probabilities of being moderately or severely underweight or stunted. While our results are in line with evidence of a positive association between milk consumption and child growth found in meta-analyses^2,20^, our country-specific estimates reflect the variation in the association on country level. A study in Western Kenya found a significant association between milk consumption and monthly height gain among children below 5 years of age^14^. Other studies showed insignificant associations for children in similar age ranges in Cambodia and Botswana^11,21^. Our results for the weight-for-height z-score and the related binary outcomes moderate and severe wasting are weakest. An explanation for the lack of a consistent effect could be that wasting captures recent nutritional problems, which may be attributable to acute food shortages, as opposed to chronic malnutrition. We could expect acute nutritional deficits to be less influenced by milk consumption, which should make up only a small part of the full diet and seems to have a stronger association with height.

Quantitatively, although not negligible, the point estimates seem rather small for the full sample. We found larger associations for children in the wealthiest quintile compared to children in the poorest quintile. This appears surprising at first. One could imagine that children in the poorest quintile with poor diets should benefit more from milk consumption than children in the wealthiest quintile with more adequate diets. However, we controlled for minimum acceptable diet in the third specification, which reduced the size of the estimated coefficients. This attenuation could indicate that the dummy for milk consumption partly proxies for a minimum acceptable diet in the second specification. Controlling for this, there was still no association for the poorest quintile. Perhaps the defined minimum acceptable diet was not sufficient for children up to 59 months of age as dietary needs increase and children in the poorest quintile lack other important nutrients that render the effect of milk moot. In addition, this indicator might not be appropriate for all specific country contexts^22^ and might not be sufficient to capture the association between infant feeding practices and child growth^23^. Another tentative explanation could be that for the poorest quintile, milk might replace other food items in the diet, even within the minimum acceptable diet, while milk might be fed in addition to a better overall normal diet in the wealthiest quintile. In our data, the proportion of children receiving the minimum acceptable diet is significantly lower in the poorest quintile (11%) than in the richest quintile (26%).

No clear picture emerged regarding differences across age groups. While associations with underweight were significant in both age groups, milk consumption was only significantly associated with a reduced probability of being stunted among children aged 24 to 59 months. Beckett *et al*. found that the effect of an additional daily ration of skimmed milk for six months had larger effects for younger cohorts of undernourished children in Indonesia than for older ones^24^. Unfortunately, we cannot compare our findings directly to the study by Beckett *et al*., as the cohorts in the older study both fall into the younger age group in our study^24^.

Our study has several limitations. Above all, our measurement of milk consumption is determined by data availability. We were not able to capture the quantity consumed or the regularity of milk consumption. Both could mediate the effect on child growth^25,26^. In addition, a 24 hour recall is not the optimal method to measure feeding practices and there is variability in daily feeding practices^18^. If this variability is larger among poorer households, for example because they cannot feed the child milk every day, this could explain the heterogeneity across wealth quintiles. In that case, we could not infer general milk consumption from the recall of milk consumption in the 24 hours before the interview. The data also does not allow us to differentiate between different types of milk, such as cow or goat milk.

Data limitations extend to covariates included in the analysis. We cannot control for several factors that may influence undernutrition in children such as perinatal health issues and maternal health during pregnancy. This is a concern if these factors also influence whether a child consumes milk and we cannot predict whether excluding any factor would lead to an over- or underestimation of the association. A robustness check of including birth weight, one indicator of maternal health during pregnancy, as an additional control variable, increases our confidence in the results. Although coefficients are estimated less precisely due to a much smaller sample size, the main results hold (see Supplementary Table S3).

Furthermore, we only provide speculations about the reason why the estimated effects were more pronounced for children from wealthier households. Although we controlled for the minimum acceptable diet, this might be insufficient. An explanation could rather lie in the exact composition of the child’s diet, which is an analysis beyond the scope of this paper and not adequately possible with the DHS.

This study contributes to the literature by providing an estimate of the association between milk consumption and child growth for a large sample of low- and middle-income countries as well as country-specific estimates. In addition, we highlight the relevance of the household’s socioeconomic status. Overall, our findings indicate that milk consumption among children aged 6 to 59 months may be beneficial for child growth. However, questions about causality remain since associations were substantially stronger for richer households, which might indicate that milk consumption is a proxy for something else and not the true cause for these differences. Furthermore, the point estimates were rather small, suggesting that milk should only be one part in a larger strategy to fight malnutrition, especially for children in the poorest households. More research is required to further investigate causality between milk consumption and child growth.

## Supplementary information

Supplementary Material.

## References

[1] Bogin, B. Milk and human development: an essay on the ‘milk hypothesis’. Vol 15 (1998).

[2] de Beer H (2012). Dairy products and physical stature: a systematic review and meta-analysis of controlled trials. Econ. Hum. Biol..

[3] Hoppe C, Mølgaard C, Michaelsen KF (2006). Cow’s Milk and Linear Growth in Industrialized and Developing Countries. Annu. Rev. Nutr..

[4] Wiley AS (2009). Consumption of milk, but not other dairy products, is associated with height among US preschool children in NHANES 1999–2002. Ann. Hum. Biol..

[5] Wiley AS (2005). Does milk make children grow? Relationships between milk consumption and height in NHANES 1999–2002. Am. J. Hum. Biol. Off. J. Hum. Biol. Assoc..

[6] Orr JB (1928). Influence of Amount of Milk Consumption on the Rate of Growth of School Children. Br. Med. J..

[7] Lampl M, Johnston FE, Malcolm LA (1978). The effects of protein supplementation on the growth and skeletal maturation of New Guinean school children. Ann. Hum. Biol..

[8] Malcolm LA (1970). Growth retardation in a New Guinea boarding school and its response to supplementary feeding. Br. J. Nutr..

[9] Grillenberger M (2003). Food Supplements Have a Positive Impact on Weight Gain and the Addition of Animal Source Foods Increases Lean Body Mass of Kenyan Schoolchildren. J. Nutr..

[10] Nhung BT (2009). Impact of milk consumption on performance and health of primary school children in rural Vietnam. Asia Pac. J. Clin. Nutr..

[11] Darapheak C, Takano T, Kizuki M, Nakamura K, Seino K (2013). Consumption of animal source foods and dietary diversity reduce stunting in children in Cambodia. Int. Arch. Med..

[12] Tumwine JK, Obala AA (2002). Nutrition status of children in Kasese district at the Uganda – Congo border. East Afr. Med. J..

[13] Choudhury S, Headey DD (2018). Household dairy production and child growth: Evidence from Bangladesh. Econ. Hum. Biol..

[14] Mosites E (2017). Child height gain is associated with consumption of animal-source foods in livestock-owning households in Western Kenya. Public Health Nutr..

[15] ICF Macro. *DHS final reports*. (2011).

[16] WHO Multicenter Growth Reference Study Group. *WHO Child Growth Standards*. *Length/height-for-age*, *weight-for-age*, *weight-for-length*, *weight-for-height and body mass index-for-age*. *Methods and development*, http://www.who.int/childgrowth/standards/technical_report/en/ (2006).

[17] WHO. *Anthro for personal computers*. (2011).

[18] Piwoz EG (1995). Potential for Misclassification of Infants’ Usual Feeding Practices using 24-Hour Dietary Assessment Methods. J. Nutr..

[19] UNICEF. *Infant and Young Child Feeding*, https://www.unicef.org/nutrition/files/Final_IYCF_programming_guide_June_2012.pdf (2012).

[20] Dror DK, Allen LH (2011). The Importance of Milk and other Animal-Source Foods for Children in Low-Income Countries. Food Nutr. Bull..

[21] Tharakan CT, Suchindran CM (1999). Determinants of child malnutrition—An intervention model for Botswana. Nutr. Res..

[22] Jones AD (2014). World Health Organization infant and young child feeding indicators and their associations with child anthropometry: a synthesis of recent findings. Matern. Child. Nutr..

[23] Reinbott A (2015). A child feeding index is superior to WHO IYCF indicators in explaining length-for-age Z-scores of young children in rural Cambodia. Paediatr. Int. Child Health.

[24] Beckett C, Durnin J, Aitchison TC, Pollitt E (2000). Effects of an energy and micronutrient supplement on anthropometry in undernourished children in Indonesia. Eur. J. Clin. Nutr..

[25] DeBoer MD, Agard HE, Scharf RJ (2015). Milk intake, height and body mass index in preschool children. Arch. Dis. Child..

[26] Huang J (2018). Early feeding of larger volumes of formula milk is associated with greater body weight or overweight in later infancy. Nutr. J..

