# Supplementary material for: Association between milk consumption and child growth for children aged 6–59 months
Source: Sci Rep. 2020 Apr 21;10:6730. doi: 10.1038/s41598-020-63647-8 (PMC7174323; doi:10.1038/s41598-020-63647-8)
Supplement: Supplementary file 1 — Supplementary Material. [file 41598_2020_63647_MOESM1_ESM.pdf]

Association between milk consumption and child growth for children aged 6-59 months

Christine Herber, Lisa Bogler, S.V. Subramanian, Sebastian Vollmer

Sample description

Table S1. Population weighted summary statistics of covariates, exposure, and outcome variables

	Whole sample	Sample of children consuming milk	Sample of children not consuming milk
Age of child	24.218	23.733	24.946
	(24.113 - 24.323)	(23.599 - 23.866)	(24.758 - 25.134)
Child is female	0.491	0.498	0.479
	(0.487 - 0.495)	(0.494 - 0.503)	(0.473 - 0.486)
Birth order: 1st	0.265	0.242	0.299
	(0.261 - 0.268)	(0.238 - 0.246)	(0.294 - 0.304)
Birth order: 2nd	0.234	0.217	0.258
	(0.230 - 0.237)	(0.214 - 0.221)	(0.252 - 0.264)
Birth order: 3rd	0.162	0.164	0.160
	(0.160 - 0.165)	(0.160 - 0.167)	(0.156 - 0.164)
Birth order: 4th or higher	0.339	0.377	0.283
	(0.335 - 0.344)	(0.372 - 0.382)	(0.276 - 0.290)
Multiple birth	0.014	0.013	0.016
	(0.014 - 0.015)	(0.013 - 0.014)	(0.014 - 0.017)
Child is currently breastfed	0.729	0.785	0.646
	(0.724 - 0.734)	(0.779 - 0.790)	(0.638 - 0.653)
Time of breastfeeding in months	15.875	16.079	15.569
	(15.817 - 15.934)	(16.004 - 16.154)	(15.474 - 15.665)
Mother's education: none	0.436	0.493	0.351
	(0.431 - 0.442)	(0.487 - 0.500)	(0.342 - 0.360)
Mother's education: primary	0.250	0.264	0.228
	(0.246 - 0.253)	(0.260 - 0.269)	(0.222 - 0.233)
Mother's education: secondary	0.257	0.209	0.331
	(0.253 - 0.262)	(0.204 - 0.213)	(0.324 - 0.338)
Mother's education: higher	0.056	0.034	0.090
	(0.053 - 0.060)	(0.031 - 0.036)	(0.084 - 0.097)
Mother's age	25.276	25.383	25.115
	(25.224 - 25.328)	(25.322 - 25.443)	(25.024 - 25.207)
Mother has partner	0.955	0.951	0.961
	(0.954 - 0.956)	(0.949 - 0.953)	(0.959 - 0.963)
Assets: radio	0.440	0.407	0.491
	(0.436 - 0.445)	(0.402 - 0.412)	(0.483 - 0.499)
Assets: TV	0.380	0.300	0.500
	(0.375 - 0.386)	(0.294 - 0.306)	(0.492 - 0.508)

Assets: bicycle	0.352	0.335	0.379
	(0.348 - 0.357)	(0.330 - 0.341)	(0.371 - 0.386)
Assets: car	0.048	0.034	0.067
	(0.046 - 0.050)	(0.033 - 0.036)	(0.063 - 0.071)
Assets: motorbike	0.155	0.137	0.182
	(0.149 - 0.160)	(0.131 - 0.143)	(0.172 - 0.193)
Assets: fridge	0.223	0.165	0.307
	(0.217 - 0.228)	(0.159 - 0.171)	(0.297 - 0.316)
Assets: phone	0.087	0.050	0.140
	(0.084 - 0.089)	(0.048 - 0.053)	(0.135 - 0.145)
Assets: electricity	0.535	0.451	0.662
	(0.529 - 0.541)	(0.444 - 0.458)	(0.655 - 0.670)
Assets: drinking water	0.283	0.224	0.372
	(0.279 - 0.288)	(0.219 - 0.230)	(0.364 - 0.380)
Assets: flush toilet	0.290	0.219	0.396
	(0.284 - 0.296)	(0.213 - 0.226)	(0.386 - 0.405)
Assets: low quality floor material	0.555	0.615	0.454
	(0.549 - 0.562)	(0.607 - 0.622)	(0.444 - 0.465)
Assets: low quality wall material	0.450	0.517	0.335
	(0.441 - 0.458)	(0.507 - 0.527)	(0.322 - 0.349)
Assets: low quality roof material	0.398	0.430	0.343
	(0.390 - 0.406)	(0.421 - 0.439)	(0.331 - 0.355)
Milk consumption	0.400	0.000	1.000
	(0.395 - 0.405)		
Z-score: weight-for-age	-1.300	-1.393	-1.161
	(-1.313 - -1.287)	(-1.409 - -1.377)	(-1.182 - -1.140)
Z-score: length-for-age	-1.702	-1.792	-1.566
	(-1.718 - -1.685)	(-1.812 - -1.772)	(-1.590 - -1.542)
Z-score: weight-for-length	-0.474	-0.520	-0.405
	(-0.485 - -0.463)	(-0.535 - -0.506)	(-0.423 - -0.388)
Stunting	0.431	0.457	0.393
	(0.427 - 0.436)	(0.451 - 0.462)	(0.386 - 0.400)
Severe stunting	0.219	0.239	0.189
	(0.215 - 0.222)	(0.234 - 0.243)	(0.184 - 0.194)
Wasting	0.134	0.141	0.122
	(0.131 - 0.137)	(0.138 - 0.145)	(0.117 - 0.128)
Severe wasting	0.045	0.049	0.040
	(0.044 - 0.047)	(0.047 - 0.051)	(0.038 - 0.041)
Underweight	0.301	0.322	0.271
	(0.297 - 0.305)	(0.317 - 0.327)	(0.264 - 0.277)
Severe underweight	0.116	0.128	0.098
	(0.113 - 0.119)	(0.124 - 0.132)	(0.095 - 0.102)

Note: Means of control, exposure, and outcome variables, population weighted. Underlying sample is the stunting sample, reduced for observations with missing exposure, outcome, and controls without minimum acceptable diet (used in model 2). 95%-Confidence intervals are given in brackets.

Development of milk consumption and undernutrition over time

Figure S1. Milk consumption

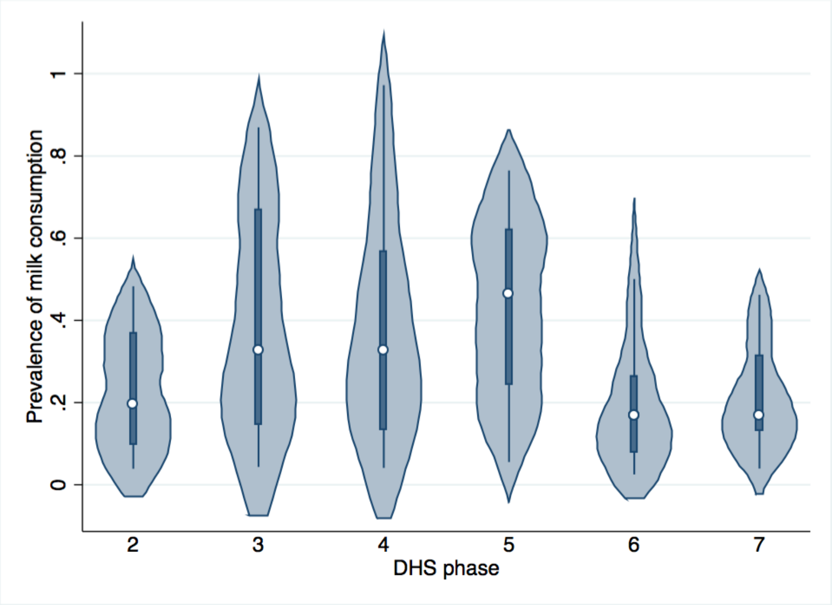

Note: Violin plots showing the prevalence of milk consumption, weighted, by DHS phase. Calculations based on stunting sample of model 2.

Figure S2. Prevalence of wasting

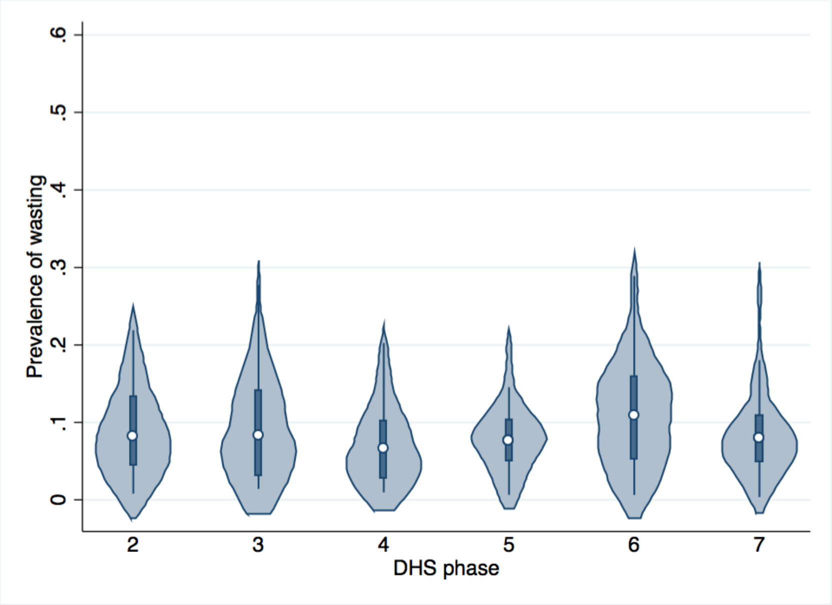

Note: Violin plots showing the prevalence of wasting, weighted, by DHS phase. Calculations based on sample of model 2.

Figure S3. Prevalence of underweight

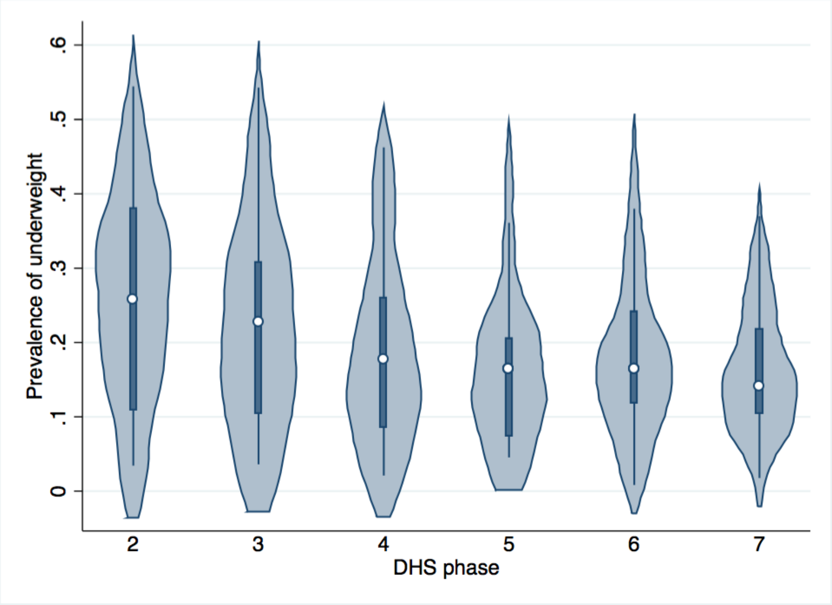

Note: Violin plots showing the prevalence of underweight, weighted, by DHS phase. Calculations based on sample of model 2.

Figure S4. Prevalence of stunting

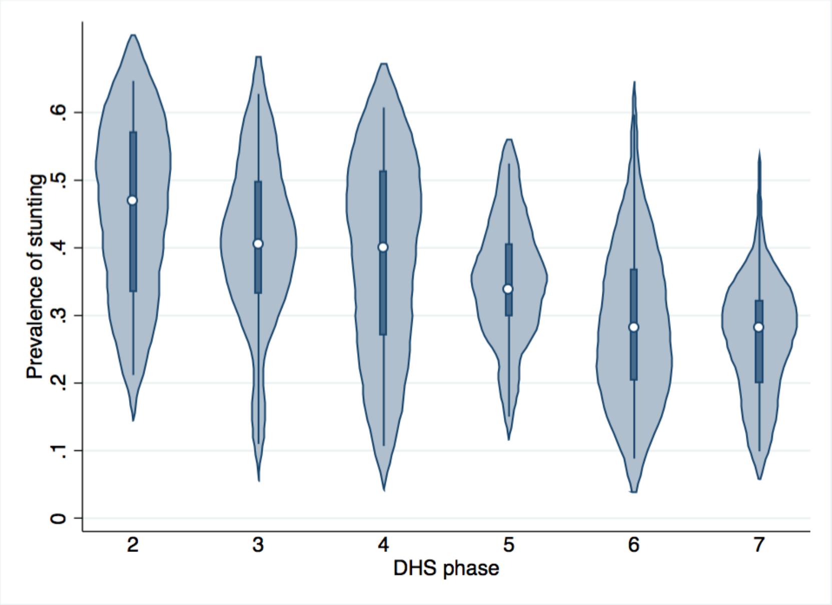

Note: Violin plots showing the prevalence of stunting, weighted, by DHS phase. Calculations based on sample of model 2.

Milk consumption and undernutrition across countries

Table S2. Milk consumption and undernutrition by country, most recent surveys

	Year of survey	Milk consumption	Prevalence of wasting	Prevalence of underweight	Prevalence of stunting	Observations
Albania	2017	0.33	0.00	0.02	0.10	409
		(0.27 - 0.40)	(-0.00 - 0.01)	(0.00 - 0.03)	(0.06 - 0.14)	
Angola	2015	0.09	0.09	0.22	0.36	1,462
		(0.07 - 0.12)	(0.07 - 0.11)	(0.20 - 0.25)	(0.32 - 0.39)	
Armenia	2015	0.16	0.02	0.02	0.13	219
		(0.11 - 0.22)	(0.00 - 0.04)	(0.00 - 0.03)	(0.09 - 0.17)	
Azerbaijan	2006	0.47	0.05	0.08	0.26	1,017
		(0.42 - 0.52)	(0.03 - 0.07)	(0.05 - 0.12)	(0.21 - 0.30)	
Bangladesh	2014	0.29	0.16	0.28	0.32	1,979
		(0.26 - 0.33)	(0.14 - 0.19)	(0.26 - 0.30)	(0.29 - 0.35)	
Benin	2017	0.13	0.08	0.19	0.27	3,014
		(0.11 - 0.15)	(0.07 - 0.09)	(0.17 - 0.20)	(0.25 - 0.28)	
Bolivia	2008	0.29	0.01	0.05	0.31	4,978
		(0.27 - 0.31)	(0.01 - 0.02)	(0.04 - 0.06)	(0.29 - 0.33)	
Brazil	1996	0.54	0.02	0.04	0.12	3,201
		(0.52 - 0.57)	(0.02 - 0.03)	(0.03 - 0.05)	(0.11 - 0.14)	
Burkina Faso	2010	0.09	0.26	0.33	0.30	1,879
		(0.07 - 0.11)	(0.23 - 0.28)	(0.30 - 0.35)	(0.28 - 0.32)	
Burundi	2016	0.05	0.09	0.28	0.49	1,723
		(0.04 - 0.06)	(0.07 - 0.10)	(0.26 - 0.31)	(0.47 - 0.52)	
Cambodia	2014	0.15	0.11	0.21	0.29	957
		(0.12 - 0.18)	(0.09 - 0.14)	(0.17 - 0.25)	(0.25 - 0.32)	
Cameroon	2011	0.18	0.06	0.17	0.35	3,532
		(0.16 - 0.20)	(0.05 - 0.07)	(0.15 - 0.18)	(0.33 - 0.37)	
Central African Republic	1994	0.07	0.09	0.26	0.45	1,855
		(0.06 - 0.09)	(0.08 - 0.11)	(0.23 - 0.28)	(0.43 - 0.48)	
Chad	2014	0.21	0.18	0.28	0.31	2,244
		(0.19 - 0.24)	(0.16 - 0.20)	(0.26 - 0.30)	(0.28 - 0.33)	
Colombia	2009	0.50	0.01	0.03	0.14	9,198
		(0.48 - 0.52)	(0.01 - 0.01)	(0.03 - 0.04)	(0.13 - 0.15)	
Comoros	2012	0.15	0.15	0.16	0.29	534
		(0.11 - 0.19)	(0.12 - 0.19)	(0.12 - 0.20)	(0.24 - 0.34)	
Congo	2011	0.28	0.10	0.15	0.20	867
		(0.24 - 0.33)	(0.07 - 0.13)	(0.11 - 0.19)	(0.16 - 0.24)	
Congo, Dem. Republic	2013	0.06	0.11	0.20	0.33	2,121
		(0.04 - 0.07)	(0.08 - 0.13)	(0.18 - 0.23)	(0.29 - 0.37)	
Côte d'Ivoire	2011	0.05	0.12	0.18	0.26	780
		(0.03 - 0.07)	(0.09 - 0.15)	(0.15 - 0.22)	(0.22 - 0.29)	
Dominican Republic	2013	0.76	0.02	0.03	0.07	1,217
		(0.72 - 0.79)	(0.01 - 0.02)	(0.02 - 0.04)	(0.05 - 0.09)	
Egypt	2014	0.18	0.12	0.08	0.21	2,713

		(0.16 - 0.20)	(0.10 - 0.14)	(0.06 - 0.09)	(0.19 - 0.23)	
Ethiopia	2011	0.20	0.15	0.27	0.37	2,260
		(0.17 - 0.23)	(0.13 - 0.18)	(0.24 - 0.30)	(0.34 - 0.40)	
Gabon	2012	0.24	0.06	0.07	0.15	502
		(0.18 - 0.29)	(0.03 - 0.09)	(0.04 - 0.09)	(0.11 - 0.18)	
Gambia	2013	0.11	0.16	0.17	0.19	881
		(0.08 - 0.14)	(0.12 - 0.20)	(0.14 - 0.21)	(0.16 - 0.23)	
Ghana	2014	0.14	0.08	0.14	0.14	689
		(0.11 - 0.17)	(0.06 - 0.11)	(0.11 - 0.17)	(0.11 - 0.17)	
Guatemala	2014	0.14	0.01	0.14	0.50	3,495
		(0.13 - 0.15)	(0.01 - 0.01)	(0.13 - 0.16)	(0.47 - 0.52)	
Guinea	2012	0.08	0.17	0.19	0.21	874
		(0.06 - 0.10)	(0.15 - 0.20)	(0.16 - 0.22)	(0.18 - 0.25)	
Guyana	2009	0.76	0.05	0.13	0.23	984
		(0.73 - 0.80)	(0.03 - 0.07)	(0.09 - 0.16)	(0.18 - 0.28)	
Haiti	2016	0.13	0.06	0.09	0.15	1,140
		(0.11 - 0.15)	(0.04 - 0.08)	(0.07 - 0.11)	(0.13 - 0.18)	
Honduras	2011	0.36	0.02	0.08	0.19	2,070
		(0.33 - 0.38)	(0.01 - 0.03)	(0.06 - 0.09)	(0.17 - 0.21)	
India	2015	0.40	0.24	0.34	0.36	61,196
		(0.40 - 0.41)	(0.23 - 0.24)	(0.34 - 0.35)	(0.35 - 0.36)	
Jordan	2012	0.42	0.01	0.01	0.09	683
		(0.35 - 0.49)	(0.00 - 0.02)	(-0.00 - 0.02)	(0.05 - 0.12)	
Kazakhstan	1999	0.97	0.02	0.04	0.14	476
		(0.95 - 0.99)	(0.01 - 0.03)	(0.02 - 0.06)	(0.10 - 0.18)	
Kenya	2014	0.45	0.05	0.13	0.27	4,326
		(0.42 - 0.48)	(0.04 - 0.06)	(0.11 - 0.14)	(0.25 - 0.29)	
Kyrgyz Republic	2012	0.23	0.02	0.03	0.15	829
		(0.19 - 0.27)	(0.01 - 0.03)	(0.01 - 0.04)	(0.11 - 0.18)	
Lesotho	2014	0.17	0.05	0.13	0.30	279
		(0.11 - 0.22)	(0.03 - 0.08)	(0.09 - 0.18)	(0.23 - 0.36)	
Liberia	2013	0.08	0.11	0.18	0.23	861
		(0.04 - 0.12)	(0.08 - 0.14)	(0.14 - 0.22)	(0.19 - 0.28)	
Madagascar	2003*	0.20	0.16	0.39	0.51	3,550
		(0.18 - 0.23)	(0.15 - 0.18)	(0.36 - 0.42)	(0.48 - 0.53)	
Malawi	2015	0.04	0.04	0.10	0.30	1,379
		(0.03 - 0.05)	(0.03 - 0.05)	(0.08 - 0.12)	(0.27 - 0.33)	
Maldives	2016	0.45	0.06	0.12	0.22	533
		(0.39 - 0.51)	(0.03 - 0.09)	(0.08 - 0.16)	(0.17 - 0.27)	
Mali	2012	0.23	0.19	0.24	0.29	1,120
		(0.20 - 0.26)	(0.17 - 0.22)	(0.21 - 0.27)	(0.26 - 0.32)	
Morocco	2003	0.57	0.10	0.10	0.24	4,533
		(0.55 - 0.60)	(0.09 - 0.11)	(0.09 - 0.11)	(0.22 - 0.26)	
Mozambique	2011	0.03	0.09	0.18	0.43	2,618

		(0.02 - 0.03)	(0.08 - 0.11)	(0.16 - 0.20)	(0.40 - 0.45)	
Myanmar	2015	0.08	0.07	0.14	0.18	1,024
		(0.06 - 0.11)	(0.05 - 0.09)	(0.12 - 0.17)	(0.15 - 0.21)	
Namibia	2013	0.08	0.12	0.12	0.16	355
		(0.05 - 0.12)	(0.08 - 0.15)	(0.08 - 0.15)	(0.12 - 0.20)	
Nepal	2015	0.46	0.14	0.26	0.32	662
		(0.42 - 0.51)	(0.11 - 0.18)	(0.22 - 0.30)	(0.28 - 0.37)	
Nicaragua	2001	0.33	0.02	0.09	0.27	3,858
		(0.30 - 0.35)	(0.01 - 0.03)	(0.07 - 0.10)	(0.25 - 0.29)	
Niger	2012	0.09	0.29	0.44	0.36	1,173
		(0.07 - 0.12)	(0.26 - 0.32)	(0.41 - 0.47)	(0.33 - 0.40)	
Nigeria	2013	0.10	0.26	0.36	0.35	5,703
		(0.09 - 0.11)	(0.25 - 0.28)	(0.35 - 0.38)	(0.33 - 0.36)	
Pakistan	2017	0.39	0.10	0.20	0.28	639
		(0.32 - 0.45)	(0.07 - 0.14)	(0.16 - 0.25)	(0.22 - 0.33)	
Paraguay	1990	0.37	0.01	0.03	0.21	1,088
		(0.33 - 0.41)	(0.00 - 0.01)	(0.02 - 0.05)	(0.18 - 0.24)	
Peru	2012	0.53	0.01	0.04	0.20	4,920
		(0.50 - 0.55)	(0.00 - 0.01)	(0.03 - 0.05)	(0.19 - 0.22)	
Rwanda	2014	0.19	0.04	0.10	0.35	1,033
		(0.16 - 0.22)	(0.02 - 0.05)	(0.09 - 0.12)	(0.32 - 0.38)	
Sao Tome and Principe	2008	0.24	0.11	0.14	0.33	1,068
		(0.20 - 0.28)	(0.09 - 0.14)	(0.11 - 0.17)	(0.29 - 0.37)	
Senegal	2017	0.23	0.11	0.15	0.15	2,649
		(0.20 - 0.25)	(0.10 - 0.13)	(0.13 - 0.17)	(0.13 - 0.17)	
Sierra Leone	2013	0.07	0.14	0.21	0.35	1,053
		(0.05 - 0.09)	(0.11 - 0.17)	(0.18 - 0.24)	(0.31 - 0.38)	
South Africa	2016	0.23	0.02	0.06	0.26	274
		(0.17 - 0.29)	(0.01 - 0.04)	(0.02 - 0.09)	(0.20 - 0.33)	
Swaziland	2006	0.31	0.03	0.06	0.30	1,177
		(0.27 - 0.35)	(0.02 - 0.04)	(0.04 - 0.07)	(0.27 - 0.33)	
Tajikistan	2017	0.37	0.08	0.09	0.13	1,134
		(0.34 - 0.41)	(0.07 - 0.10)	(0.07 - 0.11)	(0.11 - 0.15)	
Tanzania	2015	0.17	0.06	0.13	0.30	2,352
		(0.15 - 0.19)	(0.05 - 0.07)	(0.12 - 0.15)	(0.27 - 0.32)	
Timor-Leste	2016	0.07	0.29	0.37	0.39	1,134
		(0.05 - 0.09)	(0.26 - 0.32)	(0.34 - 0.40)	(0.36 - 0.42)	
Togo	2013	0.04	0.13	0.17	0.21	908
		(0.03 - 0.06)	(0.11 - 0.15)	(0.14 - 0.20)	(0.18 - 0.24)	
Turkey	1998	0.78	0.02	0.07	0.21	2,340
		(0.75 - 0.80)	(0.02 - 0.03)	(0.06 - 0.08)	(0.18 - 0.23)	
Uganda	2016	0.26	0.06	0.11	0.23	1,033
		(0.23 - 0.29)	(0.05 - 0.08)	(0.09 - 0.13)	(0.20 - 0.26)	
Uzbekistan	1996	0.80	0.12	0.14	0.39	869

		(0.76 - 0.84)	(0.09 - 0.16)	(0.10 - 0.19)	(0.35 - 0.43)	
Yemen	1991	0.37	0.14	0.31	0.57	1,288
		(0.32 - 0.41)	(0.11 - 0.17)	(0.28 - 0.34)	(0.53 - 0.60)	
Zambia	2013	0.04	0.07	0.15	0.42	5,661
		(0.03 - 0.05)	(0.06 - 0.08)	(0.14 - 0.16)	(0.40 - 0.44)	
Zimbabwe	2015	0.04	0.08	0.11	0.22	1,039
		(0.03 - 0.05)	(0.06 - 0.10)	(0.08 - 0.13)	(0.19 - 0.25)	

Note: Country-specific percentage of children consuming milk, prevalence of wasting, underweight, and stunting, weighted. 95%-Confidence intervals are given in brackets. Underlying samples are the respective wasting, underweight, and stunting samples used in model 2. Percentage of children consuming milk and number of observations are from stunting sample.

* Survey year of Madagascar for stunting is 2008.

Country-specific associations between milk consumption and undernutrition

Figure S5. Country-specific association between milk consumption and undernutrition (Adjusted Model 2)

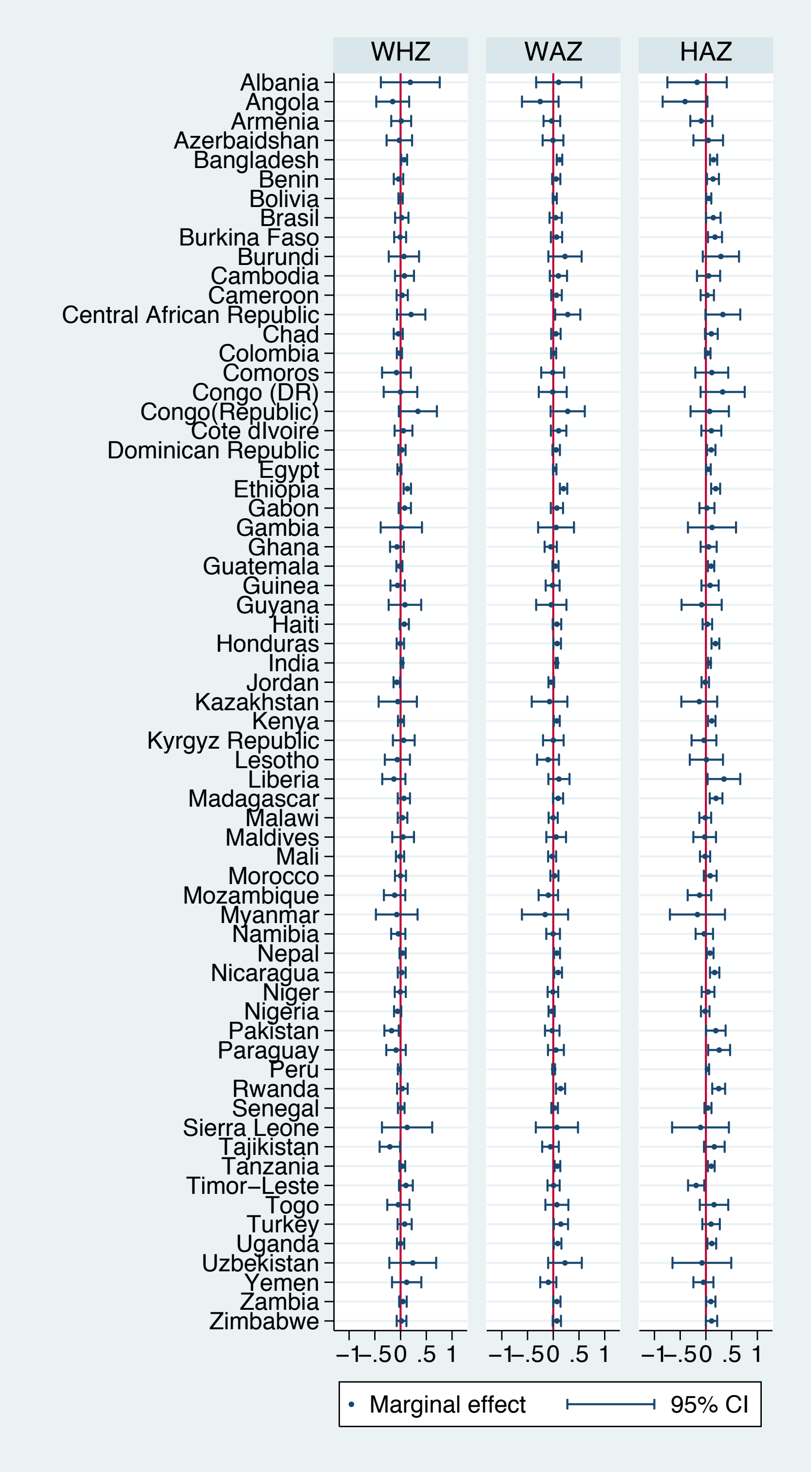

All estimates are marginal effects based on linear regressions for the second model specification (adjusted 2). Covariates: Sex of child, age of child, birth order, part of a multiple birth, child currently breastfed, duration of breastfeeding in months, education of mother, age of mother at birth, current partnership status of mother, wealth quintiles. Fixed effects on the PSU level. Standard errors are clustered on the PSU level.

Figure S6. Country-specific association between milk consumption and undernutrition (Adjusted Model 3)

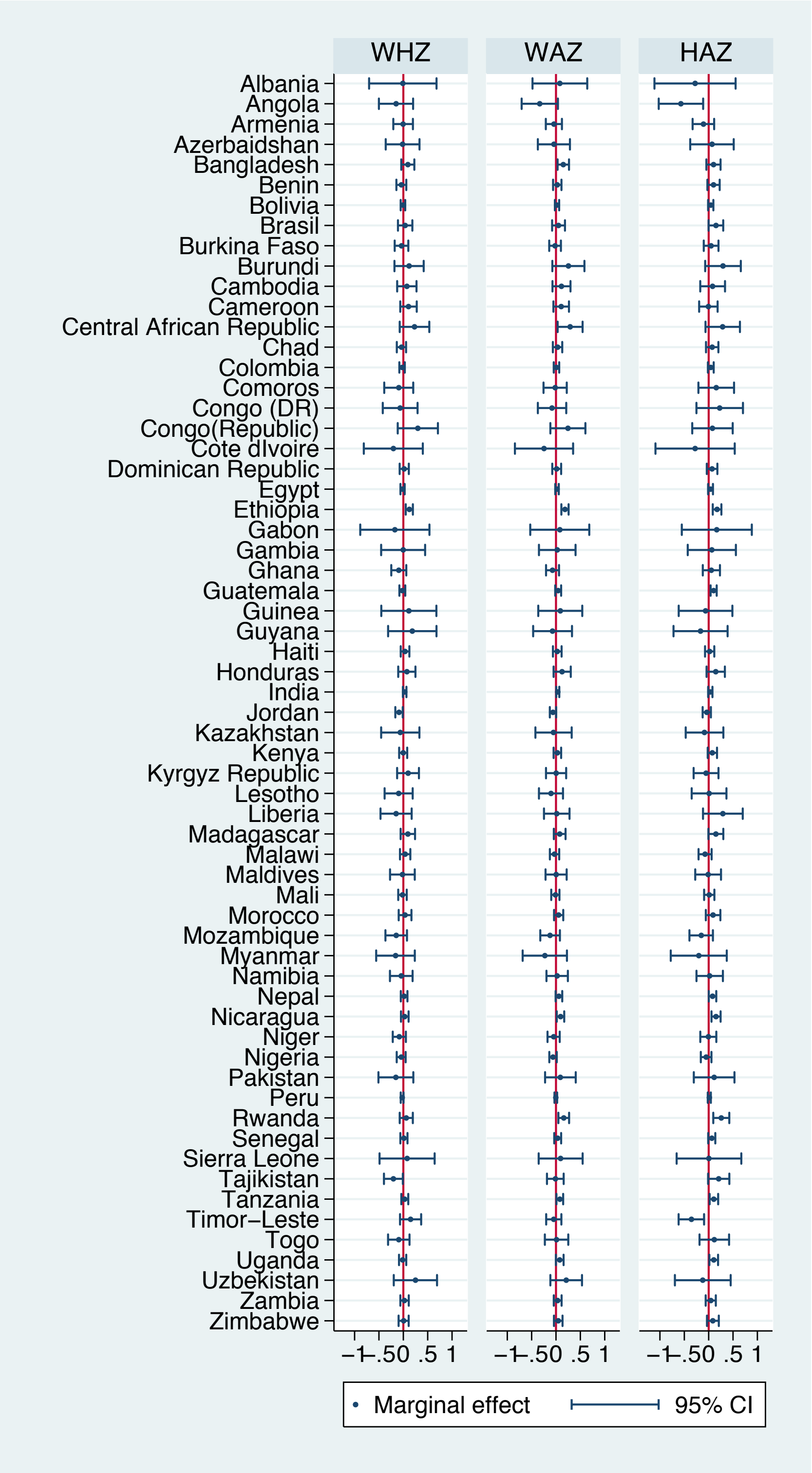

All estimates are marginal effects based on linear regressions for the third model specification (adjusted 3). Covariates: Sex of child, age of child, birth order, part of a multiple birth, child currently breastfed, duration of breastfeeding in months, education of mother, age of mother at birth, current partnership status of mother, wealth quintiles, minimum acceptable diet. Fixed effects on the PSU level. Standard errors are clustered on the PSU level.

Alternative specification with additional control variable birth weight

Table S3. Association between milk consumption and undernutrition across all wealth quintiles and age groups, extended controls

	Unadjusted			Adjusted (2)			Adjusted (3)		
**z-scores**	Weight-for-height	Weight-for-age	Height-for-age	Weight-for-height	Weight-for-age	Height-for-age	Weight-for-height	Weight-for-age	Height-for-age
Milk consumption									
	0.1566***	0.3039***	0.3063***	-0.0138	0.0335**	0.0737***	-0.0216	0.0177	0.0537***
CI 95%	[0.15,0.16]	[0.30,0.31]	[0.30,0.32]	[-0.04,0.01]	[0.01,0.05]	[0.05,0.10]	[-0.05,0.01]	[-0.00,0.04]	[0.03,0.08]
P	0.0000	0.0000	0.0000	0.2677	0.0011	0.0000	0.1384	0.0527	0.0002
N	889,710	919,973	895,599	332,362	340,678	334,797	267,973	274,637	269,584
**Moderate undernutrition**	Wasting	Under-weight	Stunting	Wasting	Under-weight	Stunting	Wasting	Under-weight	Stunting
Milk consumption (AMEs)									
	-0.0179***	-0.0595***	-0.0921***	0.0014	-0.0048***	-0.0171***	0.0035	-0.0027	-0.0113***
CI 95%	[-0.02,-0.02]	[-0.06,-0.06]	[-0.09,-0.09]	[-0.00,0.01]	[-0.01,-0.00]	[-0.02,-0.01]	[-0.00,0.01]	[-0.01,0.00]	[-0.02,-0.01]
P	0.0000	0.0000	0.0000	0.5822	0.0001	0.0000	0.2258	0.0667	0.0000
N	889,710	919,973	895,599	90,869	146,610	210,922	66,024	108,522	160,170
**Severe undernutrition**	Sev. Wasting	Sev. Under-weight	Sev. Stunting	Sev. Wasting	Sev. Under-weight	Sev. Stunting	Sev. Wasting	Sev. Under-weight	Sev. Stunting
Milk consumption (AMEs)									
	-0.0074***	-0.0237***	-0.0565***	-0.0039	-0.0054**	-0.0113***	-0.0068	-0.0023	-0.0066*
CI 95%	[-0.01,-0.01]	[-0.03,-0.02]	[-0.06,-0.05]	[-0.01,0.00]	[-0.01,-0.00]	[-0.02,-0.01]	[-0.02,0.00]	[-0.01,0.00]	[-0.01,-0.00]
P	0.0000	0.0000	0.0000	0.3149	0.0038	0.0000	0.1122	0.2344	0.0277
N	889,710	919,973	895,599	41,107	65,333	129,822	28,609	45,396	95,482

Covariates in adjusted models: Sex of child, age of child, birth order, part of a multiple birth, child currently breastfed, duration of breastfeeding in months, birthweight, education of mother, age of mother at birth, current partnership status of mother, wealth quintiles. Third specification additionally controls for a minimum acceptable diet. All adjusted models include fixed effects on the PSU level. AMEs are based on logistic regression in the first specification and on conditional logistic regression in the second and third specification. Standard errors are clustered at the PSU-level. Standard errors of adjusted models for continuous outcomes are clustered on the country level. Estimates are unweighted.

* $p < 0.05$, ** $p < 0.01$, *** $p < 0.001$

Alternative specification with interaction of milk consumption and wealth quintiles

Table S4. Wealth as mediator of association between milk consumption and undernutrition

	Adjusted (2)			Adjusted (2), no milk			Adjusted (2), interaction		
**z-scores**	Weight-for-height	Weight-for-age	Height-for-age	Weight-for-height	Weight-for-age	Height-for-age	Weight-for-height	Weight-for-age	Height-for-age
Milk consumption									
	0.0062	0.0540***	0.0809***				-0.0026	0.0320**	0.0431**
CI 95%	[-0.02,0.03]	[0.03,0.08]	[0.06,0.10]				[-0.02,0.02]	[0.01,0.05]	[0.02,0.07]
P	0.5678	0.0000	0.0000				0.7812	0.0053	0.0027
Wealth quintiles, base: quintile 1									
Quintile 2	0.0181**	0.0426***	0.0494***	0.0182**	0.0439***	0.0512***	0.0200*	0.0473***	0.0474***
CI 95%	[0.01,0.03]	[0.03,0.06]	[0.03,0.07]	[0.01,0.03]	[0.03,0.06]	[0.03,0.07]	[0.00,0.04]	[0.03,0.07]	[0.03,0.07]
P	0.0061	0.0000	0.0000	0.0064	0.0000	0.0000	0.0105	0.0000	0.0001
Quintile 3	0.0456***	0.1043***	0.1041***	0.0459***	0.1070***	0.1079***	0.0426***	0.0969***	0.0940***
CI 95%	[0.02,0.07]	[0.06,0.15]	[0.06,0.14]	[0.02,0.07]	[0.06,0.15]	[0.07,0.15]	[0.02,0.07]	[0.06,0.14]	[0.05,0.13]
P	0.0001	0.0000	0.0000	0.0002	0.0000	0.0000	0.0004	0.0000	0.0000
Quintile 4	0.0877***	0.1951***	0.1990***	0.0882***	0.1991***	0.2048***	0.0839***	0.1865***	0.1875***
CI 95%	[0.05,0.12]	[0.12,0.27]	[0.13,0.26]	[0.05,0.13]	[0.12,0.28]	[0.14,0.27]	[0.05,0.12]	[0.11,0.26]	[0.12,0.26]
P	0.0000	0.0000	0.0000	0.0000	0.0000	0.0000	0.0000	0.0000	0.0000
Quintile 5	0.1550***	0.3387***	0.3519***	0.1558***	0.3453***	0.3615***	0.1466***	0.3163***	0.3201***
CI 95%	[0.09,0.22]	[0.23,0.45]	[0.25,0.45]	[0.09,0.22]	[0.23,0.46]	[0.26,0.46]	[0.09,0.20]	[0.22,0.41]	[0.23,0.41]
P	0.0000	0.0000	0.0000	0.0000	0.0000	0.0000	0.0000	0.0000	0.0000
Milk – wealth interaction, base: no milk consumption, quintile 1									
Milk consumption & quintile 2							-0.0054	-0.0133	0.0114
CI 95%							[-0.03,0.02]	[-0.03,0.01]	[-0.02,0.04]
P							0.6629	0.1998	0.4532
Milk consumption & quintile 3							0.0112	0.0279*	0.0399*
CI 95%							[-0.02,0.04]	[0.00,0.05]	[0.01,0.07]
P							0.4104	0.0393	0.0118
Milk consumption & quintile 4							0.0133	0.0306	0.0436
CI 95%							[-0.02,0.04]	[-0.00,0.06]	[-0.00,0.09]
P							0.3664	0.0509	0.0633
Milk consumption & quintile 5							0.0224	0.0584*	0.0857***
CI 95%							[-0.02,0.06]	[0.01,0.11]	[0.05,0.12]
P							0.2460	0.0169	0.0000
N	668,463	693,376	673,177	668,463	693,376	673,177	668,463	693,376	673,177

Covariates: Sex of child, age of child, birth order, part of a multiple birth, child currently breastfed, duration of breastfeeding in months, education of mother, age of mother at birth, current partnership status of mother, wealth quintiles. All models include fixed effects on the PSU level. AMEs are based on conditional logistic regression. Standard errors are clustered on the country level. Estimates are unweighted.
$^{*}p < 0.05$, $^{**}p < 0.01$, $^{***}p < 0.001$
